# Protocol for integrating immunohistochemistry and H&E annotations with Xenium data at single-cell resolution

**DOI:** 10.1016/j.xpro.2025.104107

**Published:** 2025-09-25

**Authors:** Jun Chen, William Hartnett, Vijay S. Basava, Maximilian Lee, Baran D. Sumer, Jinming Gao, Qiang Feng

**Affiliations:** 1 Simmons Comprehensive Cancer Center, University of Texas Southwestern Medical Center, Dallas, TX 75390, USA; 2 Department of Otolaryngology, University of Texas Southwestern Medical Center, Dallas, TX 75390, USA; 3 Department of Biomedical Engineering, University of Texas Southwestern Medical Center, Dallas, TX 75390, USA; 4 Department of Cell Biology, University of Texas Southwestern Medical Center, Dallas, TX 75390, USA; 5 Department of Pharmacology, University of Texas Southwestern Medical Center, Dallas, TX 75390, USA

**Keywords:** Bioinformatics, Single Cell, Cancer, Microscopy

## Abstract

Targeted investigation of spatial transcriptomic data benefits from versatile integration of customized external information. Here, we present a protocol for embedding irregular field-of-view annotations and immunohistochemistry data into a Xenium dataset at the single-cell resolution using open-source tools. We detail steps for image registration and demonstrate how to integrate spatially aligned information into a Scanpy-compatible anndata object using a head and neck squamous cell carcinoma sample. This protocol facilitates targeted analysis of spatial transcriptomic data.

## Highlights

• Steps for image registration of Xenium data with immunohistochemistry and H&E
• Guidelines on integrating external information for single-cell analysis
• Generates Scanpy-compatible data structures for visualization

## Abstract (editor highlights)

Publisher’s note: Undertaking any experimental protocol requires adherence to local institutional guidelines for laboratory safety and ethics.

## Graphical abstract

**Figure:**
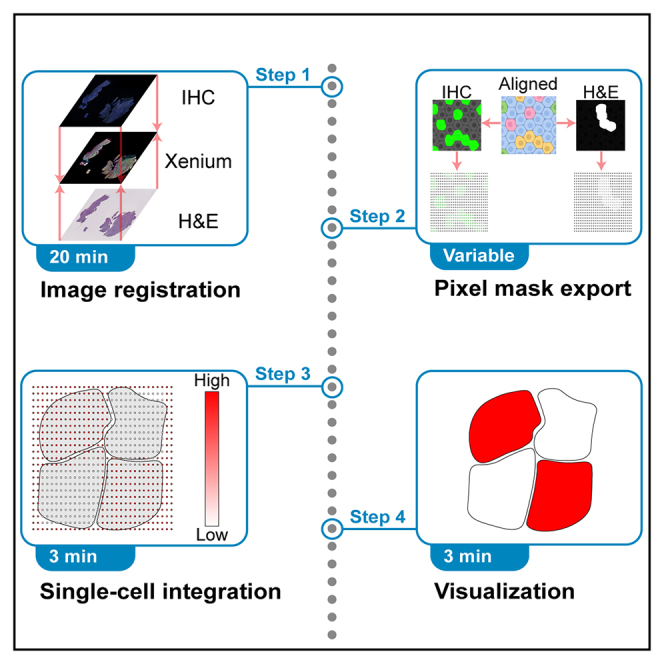

## Before you begin

Advances in spatial transcriptomics have enabled analysis of gene expression, cellular interactions, and tissue organization at single-cell resolution. *In situ* hybridization-based techniques such as MERFISH, CosMx, and Xenium allow high-resolution profiling of thousands of genes with spatial context. However, despite the extensive availability of gene-specific probes, additional context from modalities such as protein expression or expert-driven annotations is required to enhance targeted analyses. Here, we introduce a protocol to integrate information from hematoxylin and eosin staining (H&E), fluorescence immunohistochemistry (IHC), and Xenium Prime data into a unified anndata object suitable for visualization and analysis with Scanpy.^1^ Following image registration performed using Warpy^2^ *via* FIJI^3^ and QuPath,^4^ pixels and corresponding signal intensities from IHC fluorescence and binary masks of H&E annotations are assigned to individual cells based on Xenium cell segmentation boundaries. The mean signal intensity per cell is calculated and incorporated as additional observations into the anndata object. This versatile workflow facilitates downstream visualization and single-cell analyses using Scanpy and other compatible analysis plugins.

In our demonstration, we integrated human expert-labeled tumor/stroma annotations and fluorescence IHC data for monocarboxylate transporter 1 (MCT1) into the Xenium dataset at single-cell resolution. Xenium data was generated using the Xenium Prime assay with the Human 5K panel on a head and neck squamous cell carcinoma sample. IHC images were acquired using the Akoya Vectra Polaris. H&E images were obtained using the Hamamatsu NanoZoomer S60 Digital Slide Scanner.

### Innovation

This protocol advances spatial transcriptomic analysis by enabling the seamless integration of manually defined tissue annotations and immunofluorescence (IHC) data at single-cell resolution into Xenium datasets, using only open-source tools. While existing platforms like the Seurat,^5^ Giotto,^6^ and Squidpy^7^ offer comprehensive methods for spatial data integration and visualization, they do not natively support integration of IHC data or irregular annotation masks. The core innovation of our workflow is the combination of Warpy,^2^ a flexible image registration tool, with Scanpy,^1^ a Python framework for single-cell analysis. By merging these two platforms, we created a new pipeline that allows users to register IHC or H&E images, extract quantitative signals, and integrate this information directly with Xenium single-cell data. This supports context-aware spatial analysis that incorporates experimental insight from histological staining or manual annotations.

Unlike existing pipelines that rely solely on built-in spatial features, our method enables user-driven interpretation of spatial data across a wide range of tissues, imaging platforms, and experimental designs.

### Institutional permissions

Human head and neck tumor tissue was obtained from a subject who provided written informed consent prior to enrollment. The study protocol was reviewed and approved by the Institutional Review Board of the University of Texas Southwestern Medical Center (IRB# STU 092013-032).

The use of our dataset to reproduce the method described in this paper has been approved. Although this protocol does not require online data transfer, users working with their own patient-related samples may still need to obtain approval from their institutional review boards or equivalent committees.

### Set up Scanpy and Python environment

**Timing: 5–10 min**

1. Visit the Anaconda Distribution download page. Download and run the appropriate installer for your operating system.
  a. Open Anaconda Navigator after installation completes. Adding conda to path is recommended.

***Note:*** The Python version used for this protocol was 3.12.9.

2. Open terminal, and install Scanpy and its dependencies with the following code (troubleshooting 1).

conda create -n mask2sc

conda activate mask2sc

pip install numpy pandas geopandas imageio shapely rasterio affine matplotlib scanpy

conda install -c conda-forge squidpy

***Note:*** Alternatively, users can use the provided .yml file available in the GitHub repository to automatically import all dependencies. Run the following code in the terminal if the automatic method is used.

git clone https://github.com/ba-sketcvh/Mask2SC.git

cd Mask2SC

conda env create -n mask2sc --file environment.yml

conda activate mask2sc

***Note:*** The code for single-cell integration is written in Jupyter Notebook, an interactive development environment for Python. Although Jupyter Notebook is recommended for ease of use, it is not required. You may run the code in any Python IDE of your choice.

***Note:*** The image registration part with Warpy of this workflow has been validated on Windows and macOS systems, but not on Linux since Warpy official documentation indicates that their workflow has not been validated on Linux. The integration in python can be performed and has been validated on Windows, macOS, and Linux systems, including high-performance computing (HPC) clusters (e.g., Red Hat v.7.6). While the workflow was demonstrated on a system with an NVIDIA RTX A4000 GPU, GPU acceleration is not required. Users may experience slightly slower performance during image-intensive steps such as registration and visualization.

### Download QuPath, Fiji, and the Warpy extension

**Timing: 10–15 min**

3. QuPath, FIJI, and Warpy extension can be installed together using an automated method or individually. Instructions for both approaches are provided on Warpy’s website (troubleshooting 2).

***Note:*** The following analysis was performed on a computer running Windows 11; MacOS computers are also supported by Warpy.

***Note:*** Reviewing QuPath’s , Fiji’s , and Warpy’s documentation can help familiarize users with installation and exploration of their functionalities.

## Key resources table

**Table:** 

REAGENT or RESOURCE	SOURCE	IDENTIFIER
**Antibodies**		

Anti-MCT1/monocarboxylic acid transporter 1 antibody (ab85021)	Abcam	Catalog #: ab85021

**Chemicals, peptides, and recombinant proteins**		

Opal 780 reagent pack	Akoya Biosciences	Catalog #: FP1501001KT

**Deposited data**		

Example dataset	Zenodo	Zenodo: https://doi.org/10.5281/zenodo.15367950

**Software and algorithms**		

Python (v.3.12.9)	Python Software Foundation	https://www.python.org
Scanpy (v.1.11.1)	Wolf et al.^1^	https://scanpy.readthedocs.io/en/stable/index.html
Warpy	Chiaruttini et al.^2^	https://imagej.net/plugins/bdv/warpy/warpy
ImageCombinerWarpy	Chiaruttini et al.^2^	https://imagej.net/plugins/bdv/warpy/warpy
Fiji (v.1.8.0_322)	Schindelin et al.^3^	https://fiji.sc/
QuPath (v.0.5.1)	Bankhead et al.^4^	https://qupath.github.io/
Squidpy (v.1.6.5)	Palla et al.^7^	https://pypi.org/project/squidpy/
NumPy (v.2.2.6)	Harris et al.^8^	https://numpy.org/
Pandas (v.2.3.1)	McKinney^9^	https://pandas.pydata.org/
Geopandas (v.1.1.1)	Van den Bossche et al.^10^	https://geopandas.org/en/stable/
Imageio (v.2.37.0)	Klein et al.^11^	https://imageio.readthedocs.io/en/stable/
Shapely (v.2.1.1)	Gillies et al.^12^	https://shapely.readthedocs.io/en/stable/
Rasterio (v.1.4.3)	Gillies et al.^13^	https://rasterio.readthedocs.io/en/stable/
Affine (v.2.4.0)	Gillies et al.^14^	https://affine.readthedocs.io/en/latest/index.html
Matplotlib (v.3.10.3)	Hunter^15^	https://matplotlib.org/

**Other**		

Computer specifications	The computer used in this work is equipped with 16 cores, 256 GB RAM, and NVIDIA RTX A4000 GPU. ***Note:*** A computer with at least 32 GB of RAM is recommended.	

## Step-by-step method details

### Image registration using Warpy

**Timing: 20 min**

IHC or H&E images with annotations are registered to the Xenium image to accurately align resolution, pixel size, and cell boundary coordinates.

**Figure 1:**
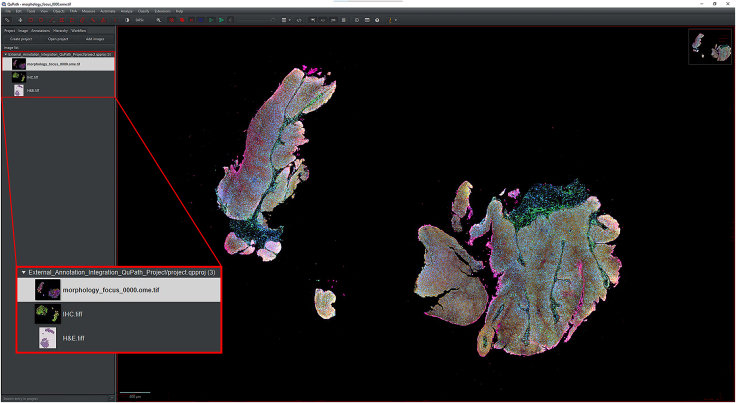
Project creation and image loading The Xenium image, IHC image, and H&E images were all uploaded into one QuPath project. Default QuPath channel colors were changed to match the original Xenium and Vectra Polaris channel colors. The red box shows a zoomed in view of images in the current project.

1. Import IHC, H&E, and Xenium images into QuPath (Figure 1).
  a. Create a new QuPath project for your images.
  b. Add your external information images and the Xenium ome.tiff into the project. Select the proper image type for each image (Fluorescent, Bright-field H&E).

***Note:*** In our provided sample dataset, H&E.tiff, IHC.tiff and morphology_focus_0000.ome.tiff were used.

***Note:*** Xenium images are in the morphology_focus folder within the Xenium output directory. Each of the four images corresponds to a different Xenium channel, but all channels will appear in QuPath regardless of which individual image is selected. For more details on the four ome.tiff images, consult 10X Genomics’ documentation on Xenium outputs.

**CRITICAL:** Ensure all images are imported using the Bio-Formats image server in QuPath. This is required for the image registration to proceed.

**Figure 2:**
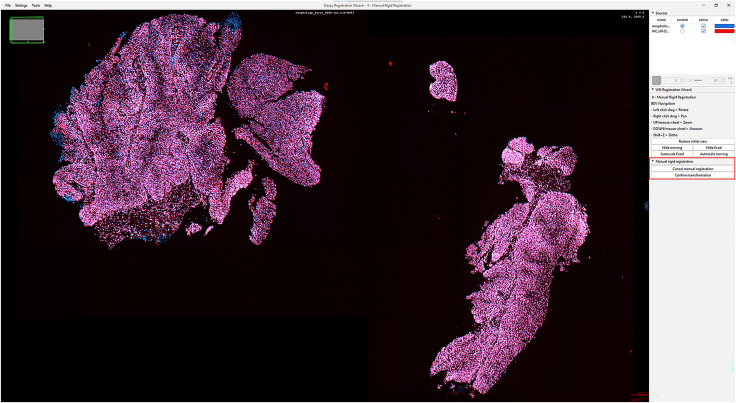
IHC and Xenium images during manual rigid registration The DAPI channels of the Xenium and IHC images are aligned with manual rigid registration. Blue: Xenium DAPI; Red: IHC DAPI. The red box highlights the “Cancel manual registration” (after clicking “Start manual rigid registration” and “Confirm transformation” buttons.

**Figure 3:**
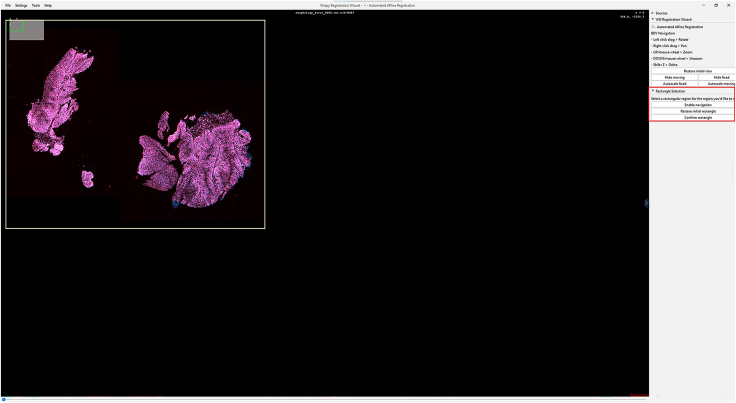
IHC and Xenium images during automated affine registration A rectangular area of focus is automatically created around the registered area. The red box shows the “Enable navigation”, “Restore initial rectangle”, and “Confirm rectangle” buttons.

**Figure 4:**
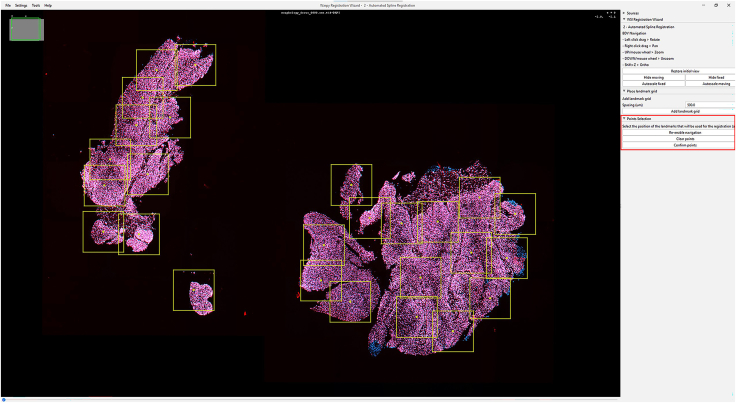
IHC and Xenium images during automated spline registration with landmarks placed Each landmark is displayed as a rectangle with a dot at the center. The red box shows the “Re-enable navigation”, “Clear points”, and “Confirm points” buttons.

**Figure 5:**
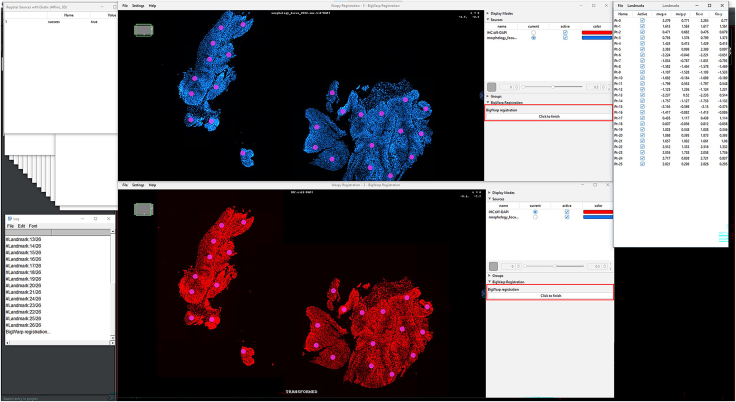
IHC and Xenium images during manual spline registration Each landmark is represented by a pink dot. Users can see how the landmarks were used during automated spline registration. Windows confirming the successful registration of each landmark appears in the upper left. A log of the current landmark being registered appears in the lower left corner. Detailed information about each set of landmarks is provided in the upper right corner. The red box highlights the “Click to finish” buttons in both images; clicking either will complete registration.

**Figure 6:**
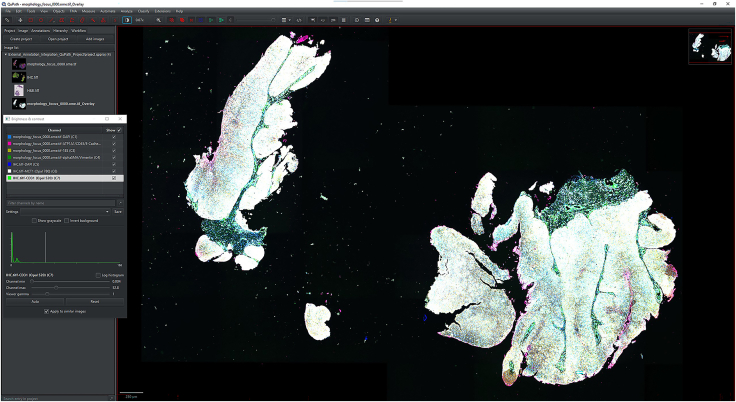
Overlayed IHC and Xenium images with all channels enabled All 4 channels from Xenium and 3 channels from IHC are displayed concurrently.

**Figure 7:**
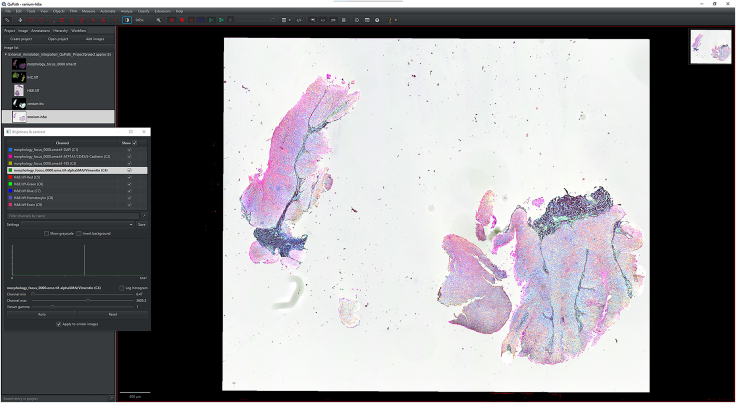
Overlayed H&E and Xenium images with all channels enabled All 4 channels from Xenium and 5 channels from H&E bright-field are displayed concurrently.

2. Register images to the Xenium image. Steps c through h follow Warpy’s protocols (troubleshooting 3).
  a. In FIJI, search for the command Create BDV Dataset [QuPath].
    i. Run this command and select your QuPath project directory in the Qupath_project box.
      ***Note:*** You can rename the dataset but keep all other settings at their default parameters.
    ii. Confirm that the channels to be registered are present (Sources -> “your project name” -> All Sources).
  b. In FIJI, search for the command QuPath – Create Warpy Registration.
    i. Run this command and set the DAPI channel of the Xenium image as the fixed source, and the DAPI channel of the IHC image or the RGB channel of the H&E image as the moving source.
      ***Note:*** A window titled “Automated WSI registration wizard” will appear. It is recommended to keep the first two boxes checked; steps 1 through 4 are optional. Check all boxes except “Show results of automated registrations (breaks parallelization)”. Keep other settings at their default values.
  c. Align the image manually with rotation and resizing during “Manual rigid registration”.
    i. Click start manual rigid registration to adjust the image.
    ii. Click Confirm transformation to confirm (Figure 2).
      ***Note:*** You may change channel colors for better visualization, which will not alter the original pixel characteristics.
  d. Define a rectangular region covering the area to be registered during “Automated Affine Registration”.
    i. Draw the smallest rectangular area that fully encloses the target region.
    ii. Click Confirm rectangle to proceed (Figure 3).
  e. During “Automated Spline Registration – Define landmarks”, place landmarks to correct warping of the moving slide.
    ***Note:*** Landmarks may overlap, and there is no maximum number of landmarks, but it is recommended to place landmarks in regions of interest and overlaps of common structures.
    i. Click Confirm points to proceed (Figure 4).
      ***Note:*** The FIJI log will confirm the coarse affine registration and landmark registration, listing each landmark as it is processed (troubleshooting 3).
  f. In “BigWarp Registration”, correct previously placed landmarks or add new ones as needed.
    ***Note:*** You can remove or reposition landmarks freely.
    i. Press Click to finish to complete registration (Figure 5).
  g. Repeat steps c through f for each image that needs to be registered.
    **CRITICAL:** The DAPI channel of the Xenium image must be selected as the fixed source to maintain native image resolution and pixel size. This step is crucial for accurately integrating pixel data.
3. Create and view registered images using ImageCombinerWarpy. These steps follow Warpy’s protocols.
  a. Open the Xenium image in the QuPath viewer.
    i. In the QuPath menu bar, navigate to Analyze -> Interactive image combiner Warpy, opening a new window.
  b. Click Choose images from project to add the other image you want to overlay.
    i. Once the image is added, select that image.
      ***Note:*** For interpolation, this protocol uses NearestNeighbor, but users may select other interpolation methods.
    ii. Click Warpy, then confirm by clicking Yes to create the overlaid image within the QuPath project (Figures 6 and 7).
      ***Note:*** The fixed image must be selected as the base image in the QuPath to create the overlaid image with ImageCombinerWarpy.
      ***Note:*** Only two files can be registered at a time. It is not possible to register an image to an already registered image.

### Export of pixel intensity from registered IHC data

**Timing: variable**

After creating the registered images, pixel intensity from IHC images can be directly exported, for integration with Xenium data.

**Figure 8:**
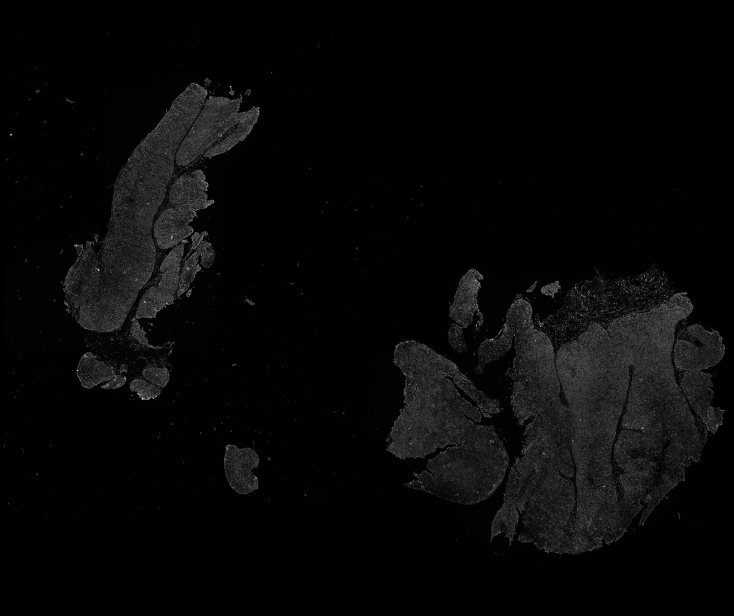
Extracted IHC channel from the IHC-Xenium registered image The displayed MCT1 IHC image is aligned with the Xenium image.

4. Extract IHC images in TIFF format, preserving original image resolution and pixel size.
  a. Click Automate in the QuPath toolbar, and then click Script editor.
    i. Open the Groovy code file or alternatively, paste the code in the following box (Figure 8).
      ***Note:*** Each channel should be extracted as its own, separate .tiff file.
      import qupath.lib.images.servers.ImageServer
      import qupath.lib.regions.RegionRequest
      import qupath.lib.images.servers.PixelType
      import java.awt.image.BufferedImage
      import javax.imageio.ImageIO
      import java.io.File
      *// Define the channels to extract (zero-based indexing: 5 = 6th channel, 6 = 7th channel)*
      def channelsToExtract = [5, 6]
      def imageData = getCurrentImageData()
      def server = imageData.getServer()
      def outputDir = buildFilePath(PROJECT_BASE_DIR, "Exported_Channels")
      mkdirs(outputDir)
      def width = server.getWidth()
      def height = server.getHeight()
      for (channelIndex in channelsToExtract) {
       println "Extracting channel ${channelIndex + 1}…"
       def region = RegionRequest.createInstance(server.getPath(), 1.0, 0, 0, width, height)
       def imgChannel = server.readRegion(region).getRaster().getSamples(0, 0, width, height, channelIndex, (double[]) null)
       def img = new BufferedImage(width, height, BufferedImage.TYPE_BYTE_GRAY)
       def raster = img.getRaster()
       raster.setPixels(0, 0, width, height, imgChannel)
       def outputFile = new File(outputDir, "Channel_${channelIndex + 1}.tiff")
       ImageIO.write(img, "TIFF", outputFile)
       println "Saved Channel ${channelIndex + 1} to ${outputFile.getAbsolutePath()}"
      }
      ***Note:*** The timing for this step depends on the number of IHC channels and computer specifications.
      ***Note:*** The .tiff images will be saved in the QuPath project directory in a folder called “Exported_Channels”.
      **CRITICAL:** Rename images to their corresponding antibodies for easier downstream analysis.
      **CRITICAL:** Ensure the extracted images have the same resolution and pixel dimensions as the original Xenium image. If needed, these channels can be uploaded into QuPath to manually change these parameters by going to the Image tab and clicking on “Pixel width” or “Pixel height”. If this is done, export the resized image as “Original pixels” in the .ome-tiff format without downsampling (troubleshooting 4).

### Creation of expert human annotations from H&E images and mask export

**Timing: variable**

After creating the overlaid image, annotations can be drawn on the H&E images to generate a binary mask for integration with Xenium data.

**Figure 9:**
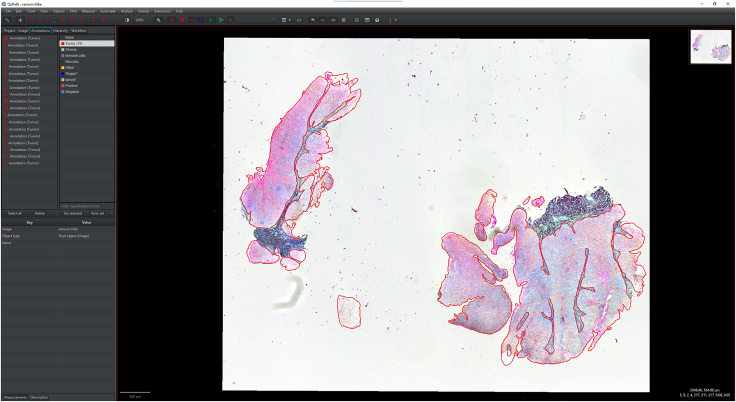
Annotations of tumor regions on the H&E-Xenium registered image Annotations (red) of the Xenium-H&E image are created with the Brush tool in QuPath.

5. Create annotations on the overlaid image according to the H&E image.
  ***Note:*** Annotations can be made using any tool, but the brush tool allows the most precise control and highest resolution for creating the mask. In this protocol, annotations were used to delineate tumor areas of the sample (Figure 9).
  a. After creating all annotations, click the Annotations tab in the Analysis panel.
    i. Click “Select all” to highlight all annotations.
    ii. Choose the most appropriate classification for the annotations.
    iii. Change its color to white and click “Set selected” to apply this classification to all annotations.
  b. Run the following code to create a binary mask of the annotations for downstream analysis.
    i. Click the Automate tab and then Script Editor to enter the code as below.
      import qupath.lib.images.servers.LabeledImageServer
      import qupath.lib.common.GeneralTools
      def imageData = getCurrentImageData()
      def server = imageData.getServer()
      int width = server.getWidth()
      int height = server.getHeight()
      double originalPixelSize = server.getPixelCalibration().getAveragedPixelSize()
      def outputFolder = buildFilePath(PROJECT_BASE_DIR, 'export')
      mkdirs(outputFolder)
      def name = GeneralTools.getNameWithoutExtension(server.getMetadata().getName())
      def pathOutput = buildFilePath(outputFolder, name + "_binary_mask.tif")
      double downsample = 1
      def labelServer = new LabeledImageServer.Builder(imageData)
       .backgroundLabel(0, 0)
       .downsample(downsample)
       .addLabel('Tumor', 255)
       .multichannelOutput(false)
       .build()
      // Save the binary mask as a TIFF file
      writeImage(labelServer, pathOutput)
      ***Note:*** Timing for this step depends on the number of annotations, image resolution, and computer specifications.
      ***Note:*** Binary masks are saved in the export folder within the QuPath project directory.
      ***Note:*** The annotations in Figure 9 are shown in red for visualization, but ensure annotation colors are white before exporting.

### Assign pixels to cells and calculate mean signal intensity at single-cell level

**Timing: 3 min**

In this step, images are loaded into Python and integrated with rasterized cell boundaries to compute the mean signal intensity per cell.

**Figure 10:**
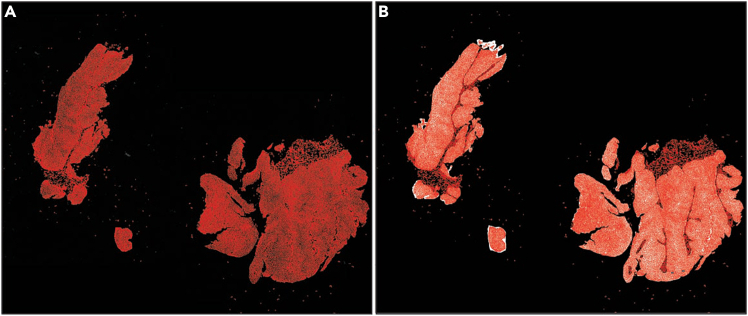
Rasterized boundaries overlaid on exported masks (A) MCT1. (B) Manual annotation derived from H&E image. Rasterized boundaries (red) are displayed over the binary mask, confirming correct alignment.

6. Execute the following Python code to generate rasterized boundaries for each cell and measure the mean signal intensity within each boundary.
  a. Set your Xenium output directory, image directory, image name, and image pixel size.
    xenium_dir = "Path_of_Xenium_output"
    img_dir = "Path_to_Image"
    channel_name = "Name_of_biomarker"
    pixel_size = 0.2125
    ***Note:*** Set the pixel size according to the Xenium image. This can be found in QuPath under the Image tab.
  b. Run the following code block without changes to read in image and cell boundary data.
    **import** os
    **import** numpy **as** np
    **import** pandas **as** pd
    **import** geopandas **as** gpd
    **from** imageio.v2 **import** imread
    **from** shapely.geometry **import** Polygon
    **from** rasterio.features **import** rasterize
    **from** affine **import** Affine
    **import** matplotlib.pyplot **as** plt
    **import** scanpy **as** sc
    **import** squidpy **as** sq
    **import** matplotlib.pyplot **as** plt
    **import** matplotlib.patches **as** patches
    # *Join the directory and file name to get the full path*
    img_path **=** os**.**path**.**join(img_dir, f'{channel_name}.tiff')
    # *Read the image*
    img **=** np**.**asarray(imread(img_path)**.**astype(np**.**float32))
    # *Join the directory and file name to get the full path*
    boundary_path **=** os**.**path**.**join(xenium_dir, "cell_boundaries.csv.gz")
    # *Read the CSV file*
    boundary_data **=** pd**.**read_csv(boundary_path)
    ***Note:*** Ensure that required Python libraries (numpy, pandas, geopandas, shapely, rasterio, matplotlib) are installed.
  c. Run the following code block without changes to assign pixels to cells and calculate the mean intensity of pixels in each cell.
    height, width **=** img**.**shape
    boundary_data["vertex_x"] **=** boundary_data["vertex_x"] **/** pixel_size
    boundary_data["vertex_y"] **=** boundary_data["vertex_y"] **/** pixel_size
    **def** make_valid_polygon(df):
     coords **=** list(zip(df["vertex_x"], df["vertex_y"]))
     **if** coords[0] **!=** coords[**-**1]:
      coords**.**append(coords[0])
     poly **=** Polygon(coords)
     **return** poly**.**buffer(0)
    boundary_polygons **=** (
     boundary_data**.**groupby("cell_id", group_keys**=False**)
    **.**apply(make_valid_polygon, include_groups**=False**)
    **.**reset_index()
    **.**rename(columns**=**{0: "geometry"}))
    boundary_gdf **=** gpd**.**GeoDataFrame(boundary_polygons, geometry**=**"geometry")
    boundary_gdf["label"] **=** range(1, len(boundary_gdf) **+** 1)
    label_to_cell_id **=** dict(zip(boundary_gdf["label"], boundary_gdf["cell_id"]))
    transform **=** Affine**.**translation(0, height) **∗** Affine**.**scale(1, **-**1)
    cell_mask **=** rasterize(
     [(geom, label) **for** geom, label **in** zip(boundary_gdf**.**geometry, boundary_gdf**.**label)],
     out_shape**=**(height, width),
     transform**=**transform,
     fill**=**0,
     dtype**=**np**.**int32)
    unique_ids **=** np**.**unique(cell_mask)
    print("Unique values in rasterized mask:", unique_ids)
    cell_mask_flipped **=** np**.**flipud(cell_mask)
    valid_pixels **=** cell_mask_flipped **>** 0
    cell_ids = cell_mask_flipped[valid_pixels]
    pixel_values **=** img[valid_pixels]
    mipc **=** pd**.**DataFrame({"label": cell_ids, "intensity": pixel_values})
    mipc **=** mipc**.**groupby("label")**.**mean()**.**reset_index()
    mipc**.**rename(columns**=**{"intensity": channel_name}, inplace**=True**)
    mipc["cell_id"] **=** mipc["label"]**.**map(label_to_cell_id)
    mipc **=** mipc[["cell_id", channel_name]]
    **CRITICAL:** Apply buffer(0) when constructing polygons to correct invalid geometries.
    ***Note:*** Users can verify results with a preliminary visualization after step 7c. Run the following code block without changes. Cell boundaries should align with the fluorescent signals in the image (Figure 10).
    print("Rendering polygon overlay on image for validation…")
    print("May take a few minutes…")
    fig, axes **=** plt**.**subplots(1, 2, figsize**=**(20, 10))
    # *First subplot: original image*
    axes[0]**.**imshow(img, cmap**=**'gray')
    axes[0]**.**set_title("Image Only")
    axes[0]**.**axis("off")
    # *Second subplot: image with boundary overlay*
    axes[1]**.**imshow(img, cmap**=**'gray')
    boundary_gdf**.**plot(ax**=**axes[1], facecolor**=**'none', edgecolor**=**'red', linewidth**=**0.1)
    axes[1]**.**set_title("Image with Boundary")
    axes[1]**.**axis("off")
    plt**.**tight_layout()
    plt**.**show()

### Visualization of IHC data and masks

**Timing: 3 min**

Mean intensity data can be mapped to its corresponding anndata object containing spatial coordinates and visualized within Python.

7. Merge the MFI data with your anndata object to align signal intensity data to individual cells. Run the following code block without changes.

# Construct full paths

h5_path **=** os**.**path**.**join(xenium_dir, "cell_feature_matrix.h5")

cells_csv_path **=** os**.**path**.**join(xenium_dir, "cells.csv.gz")

# Read the files

adata **=** sc**.**read_10x_h5(h5_path)

cells_df **=** pd**.**read_csv(cells_csv_path, index_col**=**"cell_id")

#integrate MFI information to xenium adata

cells_df **=** cells_df[cells_df**.**index**.**isin(adata**.**obs_names)]

adata**.**obsm["spatial"] **=** cells_df[["x_centroid", "y_centroid"]]**.**values

mipc **=** mipc**.**set_index('cell_id')

mipc **=** mipc**.**reindex(adata**.**obs_names)

adata**.**uns["spatial"] **=** {

   "library_id": {

    "images": {"hires": **None**, "lowres": **None**},

    "scalefactors": {

     "tissue_hires_scalef": 1.0,

     "spot_diameter_fullres": 1.0

    }

   }

  }

**for** col **in** mipc**.**columns:

 adata**.**obs[col] **=** mipc[col]

adata **=** adata[adata**.**obs**.**sort_values(channel_name, ascending**=True**)**.**index]

**Figure 11:**
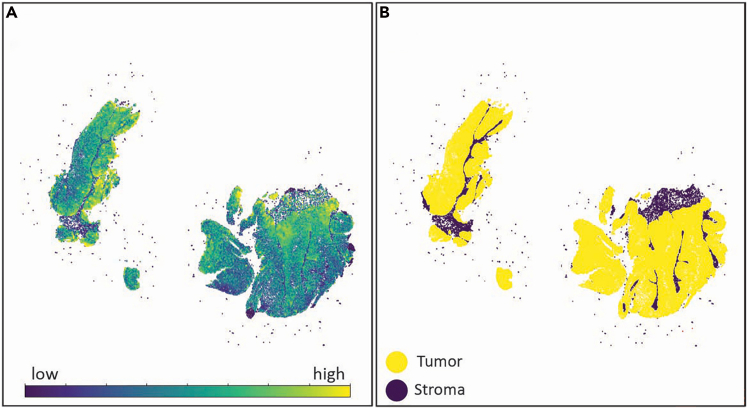
Final visualization of signal intensity per cell from external information (A) MCT1. (B) Manual annotation derived from H&E image. IHC and H&E binary masks are fully integrated with the Xenium data in Python.

8. Visualize the resulting signal using spatial coordinates (Figure 11). Run the following code block without changes unless visualization changes are needed (troubleshooting 5).

fig, ax = plt.subplots(figsize=(6, 6))

sq**.**pl**.**spatial_scatter(

 adata,

 color**=** channel_name,

 size**=**20,

 cmap**=**'viridis',

 ax**=**ax,

 img**=False**,

 vmin**=**0, *# you may change the value to threshold the displayed* image

 vmax**=**100, *# you may change the value to threshold the displayed image*

 figsize**=**(10,10)

)

ax**.**set_aspect('equal', adjustable**=**'datalim')

ax**.**set_title(channel_name)

ax**.**set_xlabel('')

ax**.**set_ylabel('')

plt.show()

***Note:*** Users can change the variables “vmin” and “vmax” for visualization purposes.

## Expected outcomes

Successful integration of IHC and/or annotations of H&E images should obtain a fully integrated dataset, enabling visualization (Figure 11). Each image channel is stored as a separate metadata column within the anndata object. For binary masks, users can create an additional metadata column by applying a threshold, splitting the spatial data into two or more groups (i.e. tumor vs stroma). Further downstream analysis can be conducted within Scanpy or, if preferred, using other Python single-cell analysis packages. Data can also be exported and converted for use within R. Thus, users can expect single-cell level spatial alignment between external information and Xenium spatial transcriptomic data.

## Limitations

Total runtime is difficult to estimate in advance due to the variations in users’ computer specifications. Processing time is influenced by image resolution, total number of segmented cells, number of channels integrated, and the computer hardware itself. Furthermore, the protocol depends on human input for several steps, which may further affect the total runtime.

This protocol depends on multiple software. Future developments of this protocol may be necessary if updates to dependent software cause unforeseen errors.

## Troubleshooting

### Problem 1

Python dependencies and packages cannot be installed.

### Potential solution

Ensure that all required packages and dependencies are listed and installed. Double-check your Python version for compatibility with each package. Using a virtual environment (e.g., venv or conda) may help manage dependencies more reliably.

### Problem 2

The Warpy extension fails to install or does not work within QuPath or FIJI.

### Potential solution

Follow Warpy’s official documentation carefully for download and installation steps. If installation of the plugin fails, or the plugin does not function within QuPath or FIJI, uninstall both programs (saving your work/completed projects) and allow the Warpy installation package to install QuPath and FIJI directly.

### Problem 3

While running Warpy, the Fiji log freezes and does not output a “successfully written” message or displays “BigWarp Registration…” in the Fiji log without confirmation of each landmark (Figure 5).

### Potential solution

Close the Warpy Registration window and repeat the image registration workflow from the beginning. If this issue persists, restart FIJI and try again.

### Problem 4

The exported binary mask does not match the expected resolution or dimensions of the Xenium image.

### Potential solution

Confirm that the binary mask was exported at the original resolution and pixel size of the Xenium image. In QuPath, verify the Image tab for “Pixel Width” and “Pixel Height” to ensure that they match the Xenium image’s metadata. If adjustments were made manually (e.g., resizing within QuPath), be sure to re-export the image as an .ome.tiff with “Original Pixels” selected and without downsampling. This ensures that the mask aligns correctly in downstream analysis.

### Problem 5

Scanpy visualization does not match the original IHC or H&E binary mask (Figure 11).

### Potential solution

Ensure that the mean intensity data is correctly aligned with the anndata object and that spatial coordinates are accurately mapped. Verify that pixel resolution and scaling have been preserved throughout the workflow. If discrepancies remain, check that the cell boundary file used for integration matches the version of the image used for annotation and export.

## Resource availability

### Lead contact

Further information and requests about executing this protocol should be directed to the lead contact, Qiang Feng (qiang.feng@utsouthwestern.edu).

### Technical contact

Technical questions about this protocol should be directed to the technical contact, Qiang Feng (qiang.feng@utsouthwestern.edu).

### Materials availability

This protocol did not generate new unique reagents.

### Data and code availability

• All original code has been deposited at https://github.com/ba-sketcvh/Mask2SC and is publicly available.
• All original data have been deposited at https://zenodo.org/records/15367950 and is publicly available.

## Acknowledgments

We thank Alex Hunter for the testing of this protocol. We thank Animesha Krishnamurthy, Shao-po Huang, Oreoluwa Onabolu, Dr. Isaac Chan, Dr. Xuechun Wang, and Dr. Yangyang Zhao for the support on acquiring Xenium data. This work was supported by the 10.13039/100000054National Cancer Institute grants R35CA294010 (J.G.), U54CA244719 (J.G.), and R01CA266146 (B.D.S.); 10.13039/100004917Cancer Prevention Research Institute of Texas grant RP220150 (J.G.); and Mendelson-Young endowment in cancer therapeutics (J.G.).

## Author contributions

Conceptualization, J.C., W.H., and Q.F.; methodology, J.C., W.H., V.S.B., and M.L.; investigation, J.C., W.H., V.S.B., and B.D.S.; writing – original draft, J.C. and W.H.; writing – review and editing, J.C., W.H., V.S.B., M.L., B.D.S., J.G., and Q.F.; supervision, B.D.S., J.G., and Q.F.

## Declaration of interests

B.D.S. and J.G. are scientific co-founders and scientific advisors of OncoNano Medicine, Inc.
